# Longitudinal changes in nutritional status during induction chemotherapy and their association with treatment outcomes in pediatric patients with newly diagnosed acute myeloid leukemia

**DOI:** 10.3389/fmed.2026.1839597

**Published:** 2026-06-01

**Authors:** Chun-lei Liu, Fan-ling Yan, Xiao-ling Li

**Affiliations:** 1 Department of Information Technology, Children’s Hospital Affiliated to Shandong University (Jinan Children’s Hospital), Jinan, Shandong, China; 2 Department of Pediatrics, Children’s Hospital Affiliated to Shandong University (Jinan Children’s Hospital), Jinan, Shandong, China

**Keywords:** induction chemotherapy, longitudinal changes, nutritional status, pediatric acute myeloid leukemia, serum albumin, treatment outcomes, treatment toxicity, weight Z-score

## Abstract

**Objectives:**

This study retrospectively analyzed longitudinal changes in key nutritional indicators—weight Z-score, BMI Z-score, and serum albumin—in children with newly diagnosed acute myeloid leukemia (AML) from the start of induction chemotherapy through 30 days post-treatment. We examined how the magnitude of decline and rate of recovery of these indicators correlated with induction remission rates, treatment-related mortality (TRM), and complication rates, with particular focus on the predictive value of weight Z-score decline for subsequent toxicities (gastrointestinal, hepatic, infectious) and remission-phase mortality. We also tested the hypothesis that greater nutritional deterioration is associated with poorer treatment outcomes, addressing limitations of conventional static assessments and informing optimal timing for early nutritional intervention.

**Materials and methods:**

This retrospective cohort study was conducted on pediatric patients with newly diagnosed AML from January 2015 to December 2024. Nutritional indicators were dynamically monitored at four time points: pre-induction (T0), chemotherapy completion (T1), 15 days post-completion (T2), and 30 days post-completion (T3). The decline magnitude (T0-T2) and recovery rate (T2-T3) of each indicator were calculated. Patients were stratified into three groups based on weight Z-score decline magnitude: mild decline (<0.5), moderate decline (0.5–1.0), and severe decline (>1.0). Induction remission, TRM, complications, and treatment toxicity were compared across the three groups. Multivariate logistic regression analysis was performed to identify the associations between nutritional indicator changes and induction treatment outcomes. Receiver operating characteristic (ROC) curves were evaluated the predictive performance of weight Z-score decline for treatment toxicities and remission-phase mortality.

**Results:**

Clinical data of a total of 62 pediatric patients (34 males, 28 females; mean age 7.3 ± 3.6 years) were analyzed. Longitudinal monitoring revealed a consistent decline - then - recovery trend for weight Z-score, BMI Z-score, and serum albumin: all indicators decreased continuously from T0 to T2, followed by gradual recovery from T2 to T3. Weight Z-score and BMI Z-score reached their nadirs at T2, while serum albumin bottomed out at T1. Patients with severe weight Z-score decline showed significantly lower remission rates (56.3% vs. 88.5% in mild and 75.0% in moderate groups) and higher TRM (18.8%) and complication rates (87.5%). They also experienced more frequent gastrointestinal, hepatic, and infectious toxicities, and higher remission-phase mortality (12.5% vs. 0.0 and 5.0%). Multivariate analysis identified weight Z-score decline > 1.0 (OR = 4.826, 95%CI: 2.673–8.725) and serum albumin decline > 10 g/L (OR = 3.715, 95%CI: 2.012–6.857) as independent risk factors for poor outcomes. ROC analysis confirmed strong predictive value of weight Z-score decline for gastrointestinal toxicity (AUC = 0.823), hepatic toxicity (AUC = 0.812), infectious toxicity (AUC = 0.854), and remission-phase mortality (AUC = 0.881).

**Conclusion:**

The nutritional status of pediatric patients with newly diagnosed AML follows a distinct decline—then—recovery longitudinal pattern during induction chemotherapy. Greater decline and slower recovery are significantly associated with worse treatment outcomes. Weight Z-score decline serves as a robust predictor of subsequent toxicities and mortality, supporting its use alongside traditional static assessments. These findings provide an evidence-based rationale for initiating nutritional support by the time chemotherapy ends.

## 1 Introduction

Acute myeloid leukemia (AML) remains one of the most common hematologic malignancies in children, with induction chemotherapy serving as the cornerstone of initial treatment (1). Outcomes such as remission rates, treatment-related mortality (TRM), and complications critically influence long-term prognosis. Nutritional status has emerged as a pivotal modifiable factor affecting chemotherapy tolerance and overall outcomes, drawing increasing attention in oncology nutrition research (2, 3). Unlike traditional cross-sectional assessments conducted at diagnosis, longitudinal monitoring captures the dynamic trajectory of nutritional changes throughout treatment, enabling identification of critical deterioration points and providing a rationale for timely nutritional interventions (4, 5).

Recent international studies have demonstrated that dynamic nutritional parameters, such as magnitude of weight loss and recovery rate, correlate closely with chemotherapy-related adverse events and prognosis in cancer patients (6). However, most domestic studies to date have focused on the association between single - time - point static nutritional indicators at initial diagnosis (e.g., BMI, serum albumin) and treatment outcomes (7, 8). There is a paucity of systematic longitudinal monitoring of nutritional indicator changes during induction chemotherapy in pediatric AML, which limits the ability to elucidate the impact of nutritional decline magnitude and recovery rate on treatment outcomes, and to validate the hypothesis that a higher degree of dynamic nutritional deterioration is associated with poorer treatment outcomes.

Weight Z-score and BMI Z-score comprehensively reflect nutritional reserves and growth status in children, while serum albumin serves as a core biomarker of visceral protein reserves. All three are clinically accessible and suitable for dynamic monitoring (9). Weight Z-score, in particular, is highly sensitive to acute nutritional changes and may serve as a practical predictor of treatment outcomes (9). In this retrospective study, we tracked these indicators longitudinally from induction initiation through 30 days post-chemotherapy, analyzed their associations with remission, TRM, and complications, and evaluated the predictive value of weight Z-score decline for toxicities and remission-phase mortality. Our goal was to validate the hypothesis that greater dynamic nutritional deterioration predicts poorer outcomes, address the limitations of static assessments, and identify an optimal window for early nutritional intervention.

## 2 Materials and methods

### 2.1 Study participants

The retrospective clinic data of pediatric patients with newly diagnosed AML from January 2015 to December 2024 was analyzed. Inclusion criteria were: (1) confirmed AML diagnosis by bone marrow aspiration, immunophenotyping, cytogenetics, and molecular biology in accordance with the *Guidelines for the Diagnosis and Treatment of Acute Myeloid Leukemia in Children* (1); (2) age 0–18 years; (3) first course of standard induction chemotherapy, with the treatment protocol determined based on the National Comprehensive Cancer Network (NCCN) Guidelines for Pediatric AML (version 2024) and risk stratification. Risk stratification was performed using the European LeukemiaNet (ELN) 2022 criteria, categorizing patients into low-risk [e.g., t (8; 21), inv. (16)], intermediate-risk (e.g., normal karyotype, NPM1 mutation without FLT3-ITD), and high-risk groups (e.g., KMT2A rearrangement, monosomy 5/7, FLT3-ITD high allelic ratio). The specific induction regimens were: DA [daunorubicin 40 mg/(m^2^·d) on days 1–3; cytarabine (Ara-C) 100 mg/(m^2^·d) on days 1–7), IA (idarubicin 12 mg/(m^2^·d) on days 1–3; Ara-C 100 mg/(m^2^·d) on days 1–7), and MA (mitoxantrone 10 mg/(m^2^·d) on days 1–3; Ara-C 100 mg/(m^2^·d) on days 1–7]; (4) no severe organ dysfunction, congenital nutritional metabolic diseases, immunodeficiency diseases, or severe gastrointestinal diseases. Exclusion criteria included: (1) acute promyelocytic leukemia (APL, FAB M3 subtype) due to distinct treatment strategies; (2) treatment withdrawal, hospital transfer, or loss to follow-up during chemotherapy or before the completion of the first post-induction assessment at T3 (30 days post-completion); (3) prior receipt of nutritional support, blood transfusion, or chemo/radiotherapy before initial diagnosis; (4) other malignancies; (5) inability to undergo anthropometric or laboratory measurements.

### 2.2 Longitudinal monitoring of nutritional indicators

Four time points were defined: T0 (baseline, within 24 h before induction), T1 (day of chemotherapy completion), T2 (15 days post-completion), and T3 (30 days post-completion). At each point, trained personnel measured height (to 0.1 cm) and weight (to 0.1 kg) using standardized equipment. BMI was calculated as weight/height^2^ (kg/m^2^). Specifically, weight was measured using a calibrated Seca 769 electronic scale (Seca, Hamburg, Germany) with children lightly clothed and without shoes. Height was measured using a Seca 417 stadiometer. BMI was calculated as weight/height^2^ (kg/m^2^). To interpret the longitudinal changes, weight and BMI Z-scores were calculated using the WHO Anthro (for children < 5 years) and WHO AnthroPlus (for children ≥ 5 years) software, which reference the 2006 WHO Growth Standards and 2007 WHO Growth Reference, respectively.

Serum albumin: fasting venous blood samples (3–5 mL) were collected, centrifuged at 3,000 rpm for 10 min and serum albumin levels were measured via automated biochemical analyzer (Siemens Advia 2,400; normal reference range of 35–55 g/L).

Decline magnitude was defined as the absolute difference T0 – T2. For weight/BMI Z-scores, decline was stratified as mild (< 0.5), moderate (0.5–1.0), or severe (> 1.0). For serum albumin, strata were mild (≤ 5 g/L), moderate (5–10 g/L), or severe (> 10 g/L). Recovery rate was defined as T3 – T2. For analysis of recovery as a categorical predictor, “rapid recovery” was defined as a recovery rate greater than the median value of the cohort (median recovery rate = 0.32), while “slow recovery” was defined as a rate ≤ the median.

### 2.3 Assessment of treatment outcomes and endpoints

Bone marrow aspiration was performed on day 28 (± 2 days) of the induction chemotherapy cycle (i.e., approximately 28 days after T0). Response was classified according to the International Working Group (IWG) response criteria for AML (1): Complete remission (CR), bone marrow blasts < 5%; absence of circulating blasts or extramedullary disease; absolute neutrophil count > 1.0 × 10^9^/L, platelet count > 100 × 10^9^/L; Partial remission (PR), decrease in bone marrow blasts to 5–25% and a decrease of at least 50% from baseline. Non-remission (NR), failure to achieve CR or PR, including persistent blasts >25%. The induction remission rate was calculated as (number of CR + PR cases/total number of cases) × 100%. Poor induction remission was defined as NR.

Treatment-related mortality (TRM) included deaths from induction start through T3 or the start of a second induction cycle (whichever occurred first) due to chemotherapy-related causes. Crucially, the longitudinal nutritional monitoring schedule (T3) was aligned with the clinical timeline. As is standard for pediatric AML protocols (e.g., NOPHO-DBH AML 2012 protocol), patients who did not achieve CR after the first induction cycle typically start a second re-induction or consolidation cycle between day 28 and day 35 post-chemotherapy. Therefore, the T3 measurement was intentionally conducted before the initiation of any subsequent chemotherapy cycle to provide a pure measure of recovery from the first cycle without the confounding effect of new treatment.

Complications (including infection, bleeding, and electrolyte disturbances) and treatment toxicities were monitored throughout induction and up to 30 days post-completion. The specific complications were defined as: infection (any CTCAE grade ≥ 2 bacterial, viral, or fungal infection requiring systemic therapy), bleeding (any CTCAE grade ≥ 2 hemorrhage not related to thrombocytopenia from disease), and electrolyte disturbance (sodium, potassium, calcium, or magnesium level requiring intervention). Toxicities were graded per *Common Terminology Criteria for Adverse Events (CTCAE) Version 5.0* (10), with a focus on definable events: gastrointestinal [nausea/vomiting (CTCAE term: Nausea, Vomiting), mucositis (Mucositis oral), diarrhea (Diarrhea)], hepatic [alanine aminotransferase increase (ALT increased)], and infectious (Febrile neutropenia, sepsis) events.

Remission-phase mortality was defined as death occurring after achieving induction remission (CR/PR) through the end of follow-up (December 2025). The median follow-up duration of (38.3 ± 9.1) months.

### 2.4 Nutritional support and mucositis prophylaxis

This study is a retrospective analysis; therefore, no standardized nutritional or mucositis prophylaxis protocol was mandated. During the study period (2015–2024), the institutional standard of care was as follows: mucositis prophylaxis, patients received standard oral care with chlorhexidine mouthwash and sodium bicarbonate rinses; Nutritional support: no prophylactic enteral or parenteral nutrition was provided. Patients were fed according to their appetite with a standard hospital diet. Dietitian consultation was triggered by a documented weight loss > 5% from baseline or serum albumin < 30 g/L. Data on the actual delivery or timing of this triggered nutritional support were not systematically recorded in the electronic medical record and thus could not be analyzed. This is a significant limitation acknowledged in the final discussion. For the present analysis, it is assumed that all patients received the same baseline level of dietary care, and any deviation was not captured.

### 2.5 Ethical statement

This study was conducted in accordance with good clinical practice guidelines and the Declaration of Helsinki. The study protocol was approved by the local ethics committee, which waived the requirement for obtaining informed consent due to the retrospective nature of the study.

### 2.6 Statistical analysis

All statistical analyses were performed using IBM SPSS 26.0 and R software (Version 4.2.1). Quantitative data were presented as mean ± standard deviation ( $\bar{x}\pm S$ ), with normality assessed by Shapiro–Wilk test. Repeated-measures analysis of variance (RM-ANOVA) with Bonferroni correction compared indicators across time points. Independent t-tests or one-way ANOVA compared intergroup differences. Categorical data are shown as counts (percentages) and compared using chi-square or Fisher’s exact test. Multivariate logistic regression (using a backward stepwise selection procedure with entry and removal *p*-values of 0.10 and 0.15, respectively) identified independent predictors of poor outcomes [composite endpoint: non-remission + TRM + severe complication (any CTCAE grade ≥ 3 complication)]. ROC analysis calculated AUC, sensitivity, specificity, and optimal cutoffs for weight Z-score decline in predicting toxicities and remission-phase mortality.

To address the potential confounding effect of pre-treatment nutritional status, an additional subgroup analysis was performed. Patients were stratified into three groups based on their baseline (T0) BMI Z-score: underweight (BMI Z-score < −1), normal weight (−1 ≤ BMI Z-score ≤ 1), and overweight/obese (BMI Z-score > 1). Induction outcomes (remission, TRM, toxicity) were then compared across these baseline groups using chi-square tests. This analysis is presented in Supplementary Table S1.

## 3 Results

### 3.1 Baseline characteristics of the study population

A total of 62 patients were included (34 males, 28 females; mean age 7.3 ± 3.6 years). The baseline characteristics are summarized in Table 1.

**Table 1:** Baseline and clinical characteristics of the 62 pediatric AML patients.

Characteristic	N (%) or $\bar{x}\pm S$
Age (years)	7.3 ± 3.6
Sex	
Male	34 (54.8%)
Female	28 (45.2%)
AML subtype (FAB)	
M0	0 (0%)
M1	6 (9.7%)
M2	29 (46.8%)
M4	13 (20.9%)
M5	14 (22.6%)
M6	0 (0%)
M7	0 (0%)
ELN 2022 risk group	
Low	15 (24.2%)
Intermediate	30 (48.4%)
High	17 (27.4%)
Induction regimen	
DA (daunorubicin/cytarabine)	35 (56.5%)
IA (idarubicin/cytarabine)	20 (32.2%)
MA (mitoxantrone/cytarabine)	7 (11.3%)
Baseline (T0) BMI Z-score	
Underweight (< −1)	9 (14.5%)
Normal (−1 to 1)	43 (69.4%)
Overweight/obese (> 1)	10 (16.1%)
Baseline (T0) weight Z-score	−0.32 ± 0.81

DA, daunorubicin/cytarabine; IA, idarubicin/cytarabine; MA, mitoxantrone/cytarabine.

### 3.2 Longitudinal changes in nutritional indicators

**Figure 1:**
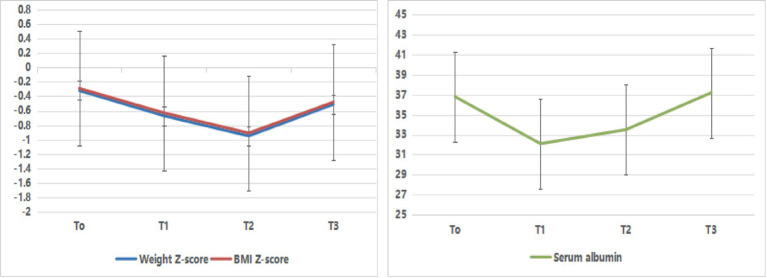
Longitudinal changes in nutritional indicators during induction chemotherapy in pediatric AML patients. Data are presented as population mean ± standard deviation ( $\bar{x}\pm S$ ). Weight Z-score (blue), BMI Z-score (red), and serum albumin (green) were measured at T0 (baseline), T1 (end of chemotherapy), T2 (15 days post-chemotherapy), and T3 (30 days post-chemotherapy). The upper and lower line segments represent the 95% confidence interval of the mean.

RM-ANOVA revealed significant differences across time points for all three indicators, with a consistent decline–then–recovery pattern (Figure 1).

Weight Z-score: decreased from T0 (−0.32 ± 0.81) to T1 (−0.67 ± 0.85), reached nadir at T2 (−0.95 ± 0.88), and recovered by T3 (−0.51 ± 0.83). BMI Z-score: decreased from T0 (−0.29 ± 0.78) to T1 (−0.63 ± 0.82), nadir at T2 (−0.91 ± 0.86), recovered at T3 (−0.48 ± 0.80). Serum albumin: The baseline serum albumin level was (36.8 ± 4.7) g/L at T0, decreased to the lowest value (32.1 ± 4.5) g/L at T1, slightly recovered to (33.5 ± 4.6) g/L at T2, and further recovered to (37.2 ± 4.8) g/L at T3.

Statistical analysis revealed significant inter-group differences (T0 vs. T1, T0 vs. T2, T2 vs. T3) in the above data. Notably, serum albumin reached its lowest point earlier (T1) than weight and BMI Z-scores (T2), and recovered faster, returning to baseline by T3. In contrast, weight and BMI Z-scores recovered more slowly and remained below baseline at T3.

### 3.3 Treatment outcomes across weight Z-score decline groups

The median recovery rate (weight Z-score change from T2 to T3) for the entire cohort was 0.32. This value was used to dichotomize “rapid” vs. “slow” recovery for subsequent analyses.

Baseline characteristics (sex, age, AML subtype, ELN risk group, chemotherapy regimen, and baseline BMI Z-score category) were comparable across the three weight Z-score decline groups (Table 2). Intergroup comparison of treatment outcomes showed a dose-dependent relationship between weight Z-score decline magnitude and treatment outcomes: A clear dose–response relationship emerged: greater decline was associated with lower remission rates, higher TRM, and more complications. The severe decline group had significantly worse outcomes across all measures, as detailed in Table 3.

**Table 2:** Baseline characteristics of patients stratified by weight Z-score decline (T0-T2).

Characteristic	Mild decline (< 0.5) (*n* = 26)	Moderate decline (0.5–1.0) (*n* = 20)	Severe decline (> 1.0) (*n* = 16)	*P*-value
Age (years)	7.1 ± 3.5	7.5 ± 38	7.4 ± 3.6	0.89
Sex				0.76
Male	14 (53.8%)	11 (55.0%)	9 (56.3%)	
Female	12 (46.2%)	9 (45.0%)	7 (43.7%)	
AML subtype (FAB)				0.68
M0	0 (0%)	0 (0%)	0 (0%)	
M1	3 (11.5%)	2 (10.0%)	1 (6.3%)	
M2	12 (46.2%)	9 (45.0%)	8 (450.0%)	
M4	5 (19.2%)	4 (20.0%)	4 (25.0%)	
M5	6 (23.1%)	5 (25.0%)	3 (18.7%)	
M6	0 (0%)	0 (0%)	0 (0%)	
M7	0 (0%)	0 (0%)	0 (0%)	
ELN 2022 risk group				0.55
Low	7 (26.9%)	5 (25.0%)	3 (18.8%)	
Intermediate	13 (50.0%)	9 (45.0%)	8 (50.0%)	
High	6 (23.1%)	6 (30.0%)	5 (31.2%)	
Induction regimen				0.82
DA	15 (57.7%)	11 (55.0%)	9 (56.3%)	
IA	8 (30.8%)	7 (35.0%)	5 (31.2%)	
MA	3 (11.5%)	2 (10.0%)	2 (12.5%)	
Baseline (T0) weight Z-score	−0.31 ± 0.79	−0.33 ± 0.82	−0.32 ± 0.80	0.99
Baseline (T0) BMI Z-score	−0.28 ± 0.75	−0.31 ± 0.80	−0.30 ± 079	0.98
Baseline serum albumin (g/L)	36.9 ± 4.5	36.7 ± 4.8	36.8 ± 4.6	0.97

DA, daunorubicin/cytarabine; IA, idarubicin/cytarabine; MA, mitoxantrone/cytarabine. All baseline characteristics were comparable across the three weight Z-score decline groups (*p* > 0.05 for all comparisons), indicating that the observed differences in treatment outcomes were not driven by pre-existing clinical or demographic disparities.

**Table 3:** Comparison of treatment outcomes among groups with different magnitudes of weight Z-score decline.

Group	*n*	Remission rate (%)	TRM (%)	Complication rate (%)
Mild decline group	26	88.5 (23/26)	0.0 (0/26)	50.0 (13/26)
Moderate decline group	20	75.0 (15/20)	5.0 (1/20)	70.0 (14/20)
Severe decline group	16	56.3 (9/16)	18.8 (3/16)	87.5 (14/16)
χ^2^	–	13.075	9.626	9.882
*P*	–	<0.05	<0.05	<0.05

TRM, treatment-related mortality.

### 3.4 Treatment toxicities and complications

Table 4 showed that incidence of CTCAE grade ≥ 2 specific toxicities (gastrointestinal: nausea/vomiting, mucositis, diarrhea; hepatic: ALT increased; infectious: febrile neutropenia, sepsis) increased stepwise with weight Z-score decline severity. Regarding the other complications (electrolyte disturbance, bleeding), no significant differences were observed across the three groups. Electrolyte disturbances occurred in 34.6% (mild), 40.0% (moderate), and 43.8% (severe) of patients (*p* = 0.52). Bleeding events (grade ≥ 2) occurred in 11.5, 15.0, and 18.8% of patients, respectively (*p* = 0.45).

**Table 4:** Comparison of CTCAE grade ≥ 2 specific toxicities among groups with different magnitudes of weight Z-score decline.

Group	*n*	Gastrointestinal (%)	Hepatic (%)	Infectious (%)
Mild decline group	26	34.6 (9/26)	23.1 (6/26)	26.9 (7/26)
Moderate decline group	20	55.0 (11/20)	40.0 (8/20)	50.0 (10/20)
Severe decline group	16	81.3 (13/16)	68.8 (11/16)	75.0 (12/16)
χ^2^		17.228	14.589	17.965
*P*-value		< 0.05	< 0.05	< 0.05

Gastrointestinal: nausea/vomiting, mucositis, diarrhea; hepatic: ALT increased; infectious: febrile neutropenia, sepsis.

Remission-phase mortality also exhibited a dose-dependent increase with weight Z-score decline severity: mild decline group (0.0%, 0/26), moderate decline group (5.0%, 1/20), severe decline group (12.5%, 2/16) (χ^2^ = 7.972, *p* < 0.05). The severe decline group had a significantly higher remission-phase mortality rate than the mild and moderate decline groups.

### 3.5 Multivariate logistic regression

**Figure 2:**
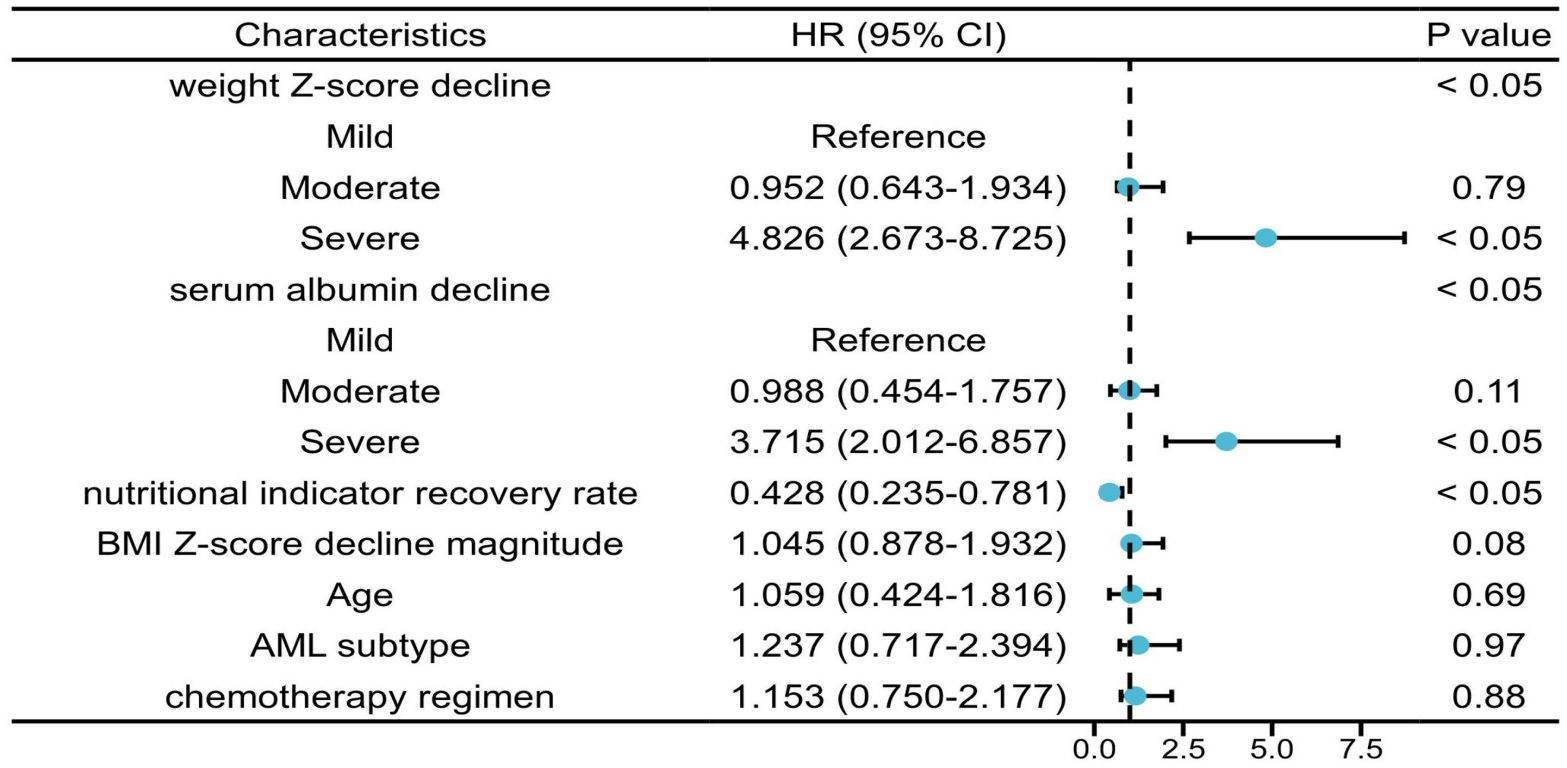
Forest plot of independent factors for adverse induction treatment outcomes in AML patients. The horizontal axis represents the odds ratio (OR) with a reference line at OR = 1 (dashed line); a square indicates the OR point estimate, and horizontal whiskers represent the 95% confidence interval (95%CI). Factors with 95%CI not crossing 1 were statistically significant (*p* < 0.05), which OR>1 = Risk factor, and OR<1 = Protective factor.

Table 5 presented the multivariate logistic regression analysis for the composite poor outcome (non-remission + TRM + severe complication). Severe weight Z-score decline (> 1.0) (OR = 4.826, 95%CI: 2.673–8.725) and severe serum albumin decline (> 10 g/L) (OR = 3.715, 95%CI: 2.012–6.857) emerged as independent risk factors. Rapid recovery (recovery rate > median of 0.32) was protective (OR = 0.428, 95%CI: 0.235–0.781). Baseline BMI Z-score category (underweight vs. normal vs. overweight/obese) was not a significant predictor (*p* = 0.34) and was removed from the final model. Age, AML subtype, and chemotherapy regimen were not significant (Figure 2).

**Table 5:** Multivariate logistic regression for poor induction outcome.

Variable	Odds ratio	95% CI	*P*-value
Weight Z-score decline > 1.0	4.826	2.673–8.725	< 0.05
Serum albumin decline > 10 g/L	3.715	2.012–6.857	< 0.05
Rapid nutritional recovery (> 0.32)	0.428	0.235–0.781	< 0.05

### 3.6 Predictive value of weight Z-score decline

**Figure 3:**
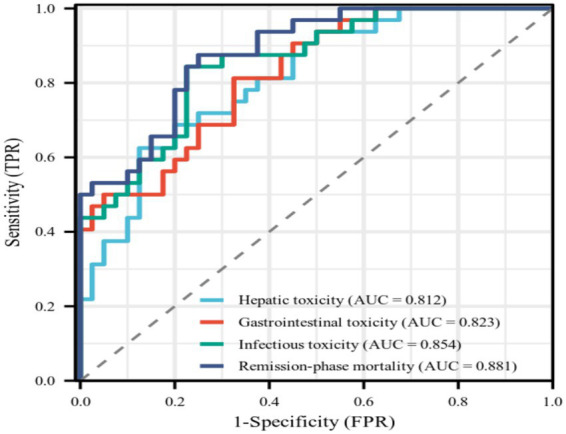
ROC curves for weight Z-score decline in predicting toxicities and remission-phase mortality.

ROC analysis showed high predictive accuracy of weight Z-score decline for all endpoints (all AUC > 0.79, *p* < 0.05) (Figure 3).

Gastrointestinal toxicity: AUC = 0.823 (95%CI: 0.756–0.890), optimal cutoff = 0.85, sensitivity = 78.6%, specificity = 76.3%.

Hepatic toxicity: AUC = 0.812 (95%CI: 0.725–0.867), optimal cutoff = 0.82, sensitivity = 75.2%, specificity = 74.1%.

Infectious toxicity: AUC = 0.854 (95%CI: 0.795–0.919), optimal cutoff = 0.88, sensitivity = 81.5%, specificity = 80.2%.

Remission-phase mortality: AUC = 0.881 (95%CI: 0.832–0.946), optimal cutoff = 0.95, sensitivity = 85.7%, specificity = 84.5%.

## 4 Discussion

Induction chemotherapy for pediatric AML induces profound cytotoxicity, systemic inflammation, and significant gastrointestinal adverse effects, all of which contribute to nutritional deterioration (5, 11, 12). While conventional cross-sectional assessments capture nutritional status only at a single time point, longitudinal monitoring reveals the dynamic trajectory of nutritional decline and recovery—information critical for timely intervention.

In this retrospective cohort study, we observed a consistent decline-then-recovery trend for weight Z-score, BMI Z-score, and serum albumin during induction chemotherapy, which is consistent with the international reports (9, 13). A novel finding was the temporal dissociation in nadirs: serum albumin bottomed out earlier (T1, chemotherapy completion) than weight and BMI Z-scores (T2, 15 days post-completion). This likely reflects albumin’s role as an acute-phase reactant, rapidly declining in response to chemotherapy-induced inflammation and mucosal injury (14). In contrast, weight and BMI Z-scores, which reflect longer-term energy reserves, decline more gradually as anorexia and catabolism persist throughout the treatment cycle (13). During recovery, albumin returned to near-baseline by T3 (30 days post-completion), probably due to resolution of inflammation and hepatic compensation, while weight-based measures lagged, indicating that nutritional rehabilitation extends beyond the immediate post-chemotherapy period.

The central finding of our study is the strong, dose-dependent association between weight Z-score decline and multiple adverse outcomes: lower remission rates, higher TRM, more complications, increased toxicities, and higher remission-phase mortality. Multivariate analysis confirmed severe weight Z-score decline (>1.0) as an independent risk factor, validating our hypothesis that greater dynamic nutritional deterioration predicts worse outcomes. Mechanistically, severe depletion of nutritional reserves likely reduces tolerance to chemotherapy, exacerbates tissue damage, and impairs innate and adaptive immune function (15–17), predisposing patients to infections and bleeding — major contributors to TRM and late mortality (18). The high predictive value of weight Z-score decline for toxicities and mortality (AUC up to 0.881) supports its use as a practical, early warning biomarker.

It is critical to acknowledge that while our data demonstrate a robust association, inferring direct causality is limited by the study’s retrospective design. The documented association between decline during the first chemotherapy cycle and outcomes over the entire follow-up period could be confounded by unmeasured variables, such as inherent disease aggressiveness or subclinical inflammatory status at diagnosis. For instance, patients with high-risk cytogenetics may experience both more severe chemotherapy-induced toxicity and a higher relapse rate, independently of nutritional status. Although we included ELN risk group in the multivariate model and it was not a significant predictor, residual confounding cannot be excluded. Furthermore, data on nutritional status at the exact time of remission-phase mortality events were not available; these late deaths could be driven more by disease relapse than by residual nutritional deficit from the first cycle. Therefore, our results should be interpreted as hypothesis-generating, providing a strong rationale for prospective interventional trials, rather than definitive proof of a causal chain from early nutritional decline to late mortality.

Severe serum albumin decline (>10 g/L) also emerged as an independent risk factor, consistent with prior studies (19, 20). As a marker of visceral protein stores, albumin reflects the balance between synthesis and catabolism. Its sharp decline indicates inadequate protein reserves to support mucosal repair, immune function, and tissue recovery, thereby compounding chemotherapy-induced damage (19). Interestingly, BMI Z-score decline did not remain significant in multivariate analysis, likely because BMI is less sensitive than weight Z-score to acute changes in children. This reinforces the choice of weight Z-score as the preferred metric for longitudinal monitoring.

Another important observation was that faster nutritional recovery independently predicted better outcomes. Rapid recovery likely reflects greater physiological resilience and capacity for tissue repair, which can mitigate chemotherapy-related adverse events and improve prognosis (21). This finding underscores the importance of not only minimizing decline but also actively supporting recovery. Based on our data, we propose initiating nutritional support before or at the time of chemotherapy completion (T1)—when serum albumin reaches its nadir and weight Z-score begins its steep decline. Patients with weight Z-score decline ≥ 0.5 or serum albumin decline ≥ 5 g/L by T1 should be prioritized for targeted interventions such as enteral nutrition or oral supplements. This approach aligns with international recommendations (21, 22) and provides a practical, evidence-based trigger for early nutritional support in pediatric AML.

Finally, our study underscores a critical gap in clinical practice. The lack of a standardized, protocol-driven nutritional intervention during the study period (with support triggered only after significant weight loss) means we were effectively observing the natural history of nutritional decline. This strengthens our finding that weight Z-score decline is a powerful prognostic marker, as it was not mitigated by a systematic intervention. It also highlights the urgent need for prospective trials testing early, individualized nutritional support, starting at chemotherapy completion (T1), particularly in patients who show a decline of ≥ 0.5 in weight Z-score by this early timepoint.

## 5 Study limitations

Several limitations warrant consideration. First, this is a single-center study with a modest sample size, which may limit generalizability. Second, a major limitation is the absence of a standardized protocol for nutritional support and mucositis prophylaxis. The retrospective and inconsistent application of triggered nutritional support prevents us from assessing its impact and is a potential source of unmeasured confounding. Third, we did not measure inflammatory markers (e.g., C-reactive protein, interleukin-6), gut microbiota, or dietary intake, precluding mechanistic insights. Fourth, we lacked data on nutritional status at the time of late events (e.g., remission-phase mortality) and the exact timing of second chemotherapy cycles relative to T3, although protocol schedules suggest T3 preceded a second cycle. Fifth, follow-up was relatively short (median 38.3 months), and long-term survival outcomes (e.g., 5-year OS, EFS) were not assessed

## 6 Conclusion

In this single-center retrospective study, nutritional status in children with newly diagnosed AML followed a distinct decline–then–recovery trajectory during induction chemotherapy. Greater decline and slower recovery were strongly associated with poorer treatment outcomes. Weight Z-score decline proved to be a simple, clinically accessible predictor of subsequent toxicities and mortality, complementing traditional static assessments and offering a rationale for early nutritional intervention starting at chemotherapy completion. Future multicenter studies with larger cohorts, mechanistic biomarkers, and interventional trials are needed to refine and validate these findings.

## Data Availability

The raw data supporting the conclusions of this article will be made available by the authors, without undue reservation.
