# Supplementary material for: Longitudinal changes in nutritional status during induction chemotherapy and their association with treatment outcomes in pediatric patients with newly diagnosed acute myeloid leukemia
Source: Front Med (Lausanne). 2026 Jun 1;13:1839597. doi: 10.3389/fmed.2026.1839597 (PMC13265345; doi:10.3389/fmed.2026.1839597)
Supplement: Supplementary file 1 [file Supplementary_file_1.doc]

Supplementary Table S1. Induction Outcomes Stratified by Baseline (T0) BMI Z-score Category

| Baseline BMI Z-score Category | n | remission rate  (CR + PR) (%) | TRM (%) | Any Grade ≥ 3 Toxicity (%) |
| --- | --- | --- | --- | --- |
| Underweight (< -1) | 9 | 77.8 (7/9) | 11.1 (1/9) | 77.8 (7/9) |
| Normal (-1 to 1) | 43 | 81.4 (35/43) | 7.0 (3/43) | 62.8 (27/43) |
| Overweight/Obese (> 1) | 10 | 80.0 (8/10) | 10.0 (1/10) | 70.0 (7/10) |
| *P*-value |  | 0.92 | 0.78 | 0.45 |

Note: TRM = treatment-related mortality. No statistically significant association was found between baseline BMI Z-score category and any induction outcome, suggesting the dynamic change (decline) is a more critical prognostic factor than pre-treatment nutritional status alone.
